# Sickle Cell Anemia Screening in Newborns and Analysis of Haplotypes in Patients from Santiago Island, Cape Verde

**DOI:** 10.1155/2024/1687917

**Published:** 2024-08-27

**Authors:** Ariana Freire, Laura Charola-Ramos, Elisa González-Guerra, João Gonçalves, Vanusa Rocha, Vera Afreixo, Enrique Martínez-Carretero, José M. Raya

**Affiliations:** ^1^ University of Cape Verde (UniCV), Santiago, Cape Verde; ^2^ University of La Laguna (ULL) Institute of Tropical Diseases and Public Health, Tenerife, Spain; ^3^ University Hospital Dr. Agostinho Neto (UHAN), Praia, Cape Verde; ^4^ University of Aveiro (UA), Aveiro, Portugal; ^5^ University of La Laguna Hospital University of Canary Island, Tenerife, Spain

## Abstract

Sickle cell anemia (SCA) results from a mutation in the *β*-globin gene, leading to the production of mutant hemoglobin, known as hemoglobin S (HbS). Despite being a genetic disorder, the phenotype of SCA can be influenced by the level of fetal hemoglobin (HbF), which is associated with beta S-globin haplotypes. In this study, we conducted newborn screening (NBS) using samples collected from umbilical cord blood in two hospitals on Santiago Island, Cape Verde. In newborns, HbS was detected using high-performance liquid chromatography (HPLC) on dried blood spot, with confirmation through polymerase chain reaction (PCR) and restriction fragment length polymorphism (RFLP). In addition, we assessed the hematological and clinical characteristics of a second population group consisting of patients diagnosed with SCA. Haplotype determination was performed on both newborns with HbS and patients with SCA. Beta S-globin haplotypes were determined using PCR-RFLP. Hematological values were analyzed using standard methods. Out of 346 newborns, 21 (6%) were carriers of the sickle cell trait (HbAS) while none were identified as homozygous for sickle cell disease (HbSS). Among both groups of individuals, four haplotypes were identified: Senegal, Arabi-Indian, Bantu, and Benin. The Senegal haplotype was the most prevalent, possibly reflecting the ethnic origin of the mutations observed. Hematological values did not differ significantly among haplotypes. However, higher levels of HbF were associated with better hematological values. These findings suggest a positive impact of elevated HbF levels on reducing the severity of SCA. Finally, we demonstrated how the combination of technics, HPLC and molecular analysis, provided a consistent and reproducible results that can be used for NBS for SCA.

## 1. Introduction

Sickle cell anemia (SCA) is a type of hemoglobinopathy and one of the most common severe monogenic disorders in the world [1]. Specifically, SCA is a genetic disorder resulting from the substitution of adenine by thymine, which leads to the substitution of glutamic acid for valine at position 6 in the beta-globin molecule. This mutation gives rise to hemoglobin S (HbS) instead of normal adult hemoglobin (HbA). The inheritance of HbS can be heterozygous (HbAS), known as the sickle cell trait, or homozygous (HbSS), which results in SCA [2, 3]. With different solubility and upon deoxygenation, HbS forms large polymers that affect the shape and function of erythrocytes, causing them to become sickle cells (SCs) [4]. These sickle cells have a short lifespan and are easily destroyed by hemolysis, leading to hemolytic anemia. In addition, these deformed red blood cells are prone to adhere to vessels, causing vaso-occlusion phenomena. This disease leads to several complications such as pain crises, leg ulcers, infections, priapism, splenic infarction, or even stroke [5, 6].

Most countries in the Sub-Saharan Africa region do not have newborn screening programs for SCA [7, 8]. Cape Verde is no exception to this reality. Located in the Sub-Saharan Africa region, Cape Verde has around 570,000 inhabitants and a birth rate of 10,544 live births per year. These islands were discovered uninhabited by the Portuguese in 1460 [9]. The settlement process of Cape Verde began around 1462 with African and European people involved in the Atlantic slave trade. This process resulted in a highly mixed population, with the potential for a great diversity of hemoglobinopathy alleles within the population [10, 11]. Preliminary studies suggest that the allelic frequency of HbS in Santiago Island is 5% [12].

Usually, the first symptoms of SCA begin after 6 months of age, coinciding with the hemoglobin (Hb) transition from HbF to HbA, or in this case, specifically, the switch is from HbF to HbS [13]. Early diagnosis of SCA has a significant impact on patients' lives as it allows timely treatment, such as the administration of prophylactic penicillin and vaccination, reducing mortality in children under 5 years old. According to the World Health Organization (WHO), approximately 7% of the world's population carries hemoglobin disorders, and between 300,000 and 500,000, infants are born each year with the severe homozygous form [14]. Among these, more than 200,000 are Africans with sickle cell disease (SCD), accounting for 5% of overall mortality in children under 5 years old on this continent [14]. The sub-Saharan Africa region is considered an epicenter of SCA, with more than 75% of babies born with SCD worldwide each year [15, 16]. Furthermore, in Cape Verde, around 4,030 blood transfusions are performed annually in national hospital services. Individuals with SCA may constitute a target group for these transfusions, as they are considered within the treatment of these patients in crisis [17, 18]. However, even taking into account all these circumstances, in Cape Verde, there is no newborn screening program for SCA or well-designed diagnostic system for this disease.

Nevertheless, although SCA is a genetic disease, the severity of complications can be modulated by factors such as fetal hemoglobin (HbF) levels, which are related to beta S-globin haplotypes. Haplotypes are defined by polymorphic DNA sequences in the *β*-globin gene cluster [19–21]. There are five common haplotypes associated with SCA, named according to the geographic areas where they predominate: Senegal (SEN), Benin (BEN), Bantu (BAT) (or Central Africa Republic, CAR), Cameroon (CA), and Arabic-India (AI) [22, 23]. Arabic-Indian and Senegal haplotypes are associated with higher Hb levels, nearly 20% and 10%, respectively, contributing to a milder disease. Meanwhile, the Bantu haplotypes exhibit a more severe clinical phenotype, primarily attributed to lower levels of HbF, approximately 5% [19, 24]. The classification of patients' haplotypes provides prognostic information, and also, it helps better understand the genetic variability associated with the disease and its distribution in different populations [19–21].

The aims of our study were to determine the prevalence of HbS in the Santiago Island population and analyze the association between haplotypes and clinical phenotypes of sickle cell patients.

## 2. Materials and Methods

### 2.1. Sampling

A total of 360 samples from newborns were collected between August 24th and November 6th, 2019. Ultimately, 346 samples were included in the study, with 14 being deemed unsuitable. Of these, 60.7% were from University Hospital Dr. Agostinho Neto (UHAN), while 39.3% were from Hospital Saint Rita Vieira (HRSN). The majority of parents (95.9%) were residents of the Santiago Island. Among the 346 samples analyzed, 169 were from girls and 177 from boys. Children included in this study were Cape Verdean up to the second generation, meaning that their grandparents should be Cape Verdean. Nonadequate samples, such as those that were coagulated or insufficient, were excluded.

Samples from patients with SCA were collected at the Blood Bank of UHAN. This included patients diagnosed with SCA who were being treated at UHAN. This group consisted of 33 patients, with a median age of 24 years, of whom 19 were males and 14 females. The age of diagnosis for SCA in these patients was elevated, around 10 years old. Patients who had received a blood transfusion less than 3 months before the sampling date were excluded.

In addition to blood samples, patients were weighed and measured for height, and a questionnaire was given to all participants.

Blood samples from newborns and patients were collected in ethylenediaminetetraacetate (EDTA) tubes. From the EDTA sample tubes, a portion of the blood sample was placed on paper filters (Whatman G003) to obtain dried blood spots (DBSs) for a subsequent analysis by high-performance liquid chromatography (HPLC). Another portion of the samples was stored at −20 degrees Celsius for a molecular analysis. Laboratory analyses were conducted at the Institute of Tropical Diseases and Public Health of the Canary Islands, University of La Laguna (Tenerife, Spain), University of Cape Verde (UniCV), and UHAN.

### 2.2. Laboratory Analyses

All specimens, including newborns and patients with SCA, were screened using the Bio-Rad D-10 machine, which employs ion-exchange HPLC principles [12, 25]. Positive samples for HbS identified through HPLC were subsequently confirmed using molecular methods, including ARMS-PCR (amplification refractory mutation system-polymerase chain reaction) and RFLP (restriction fragment length polymorphism). The hematological analysis was performed through patients' blood counts, utilizing the Sysmex K21N hematology analyzer at UHAN.

### 2.3. DNA Extraction

DNA was extracted from frozen blood using the FastPrep® 24 5G system (MP Biomedicals™) with Lysing Matrix A® (MP Biomedicals™). The lysing buffer contained 0.1 M NaCl, 10 mM Tris-HCl pH 8.0, 25 mM EDTA pH 8.0, and 0.5% SDS pH 7.2. Briefly, 500 *µ*l of lysing buffer and 300 *µ*l of whole blood were added to the lysing matrix A and mixed by inversion. After a few minutes' incubation, the mixture was processed for 40 seconds at a speed setting of 6.0 m/s using the FastPrep24.

### 2.4. HbS Determination

#### 2.4.1. PCR Amplification

The ARMS has been developed for diagnosis of hemoglobinopathies, and it allows the detection of known point mutations [26–29]. Amplification of DNA was carried out using the previously described primers [29, 30]. Amplification reactions were performed in a final volume of 50 *µl* with 2 *µ*l of DNA and a master mix containing buffer solution 1X (+MgCl2), 0.25 mM of each dNTP's, 0.4 *µ*M of each primer, and 0.025 U/*µ*l of VWR® Taq DNA polymerase. For primers S1/S2, amplification of DNA was carried out using the following program: initial denaturation for 10 minutes, 30 cycles of 94°C for 30 s, 53°C for 30 s, and 72°C for 30 s, and final elongation for 7 minutes. For primers SN1/SN2 and SMUT1/SN2, amplification was performed as follows: initial denaturation for 5 minutes, 30 cycles of 94°C for 30 s and 69°C for 45 s, and final elongation for 7 minutes.

PCR were analyzed on 1.5% or 1.8% agarose gel electrophoresis stained with REALSAFE Nucleic Acid Staining®.

#### 2.4.2. Restriction Enzyme Digestion

Digestion of S1/S2 amplified products was carried out with the DdeI restriction enzyme (Promega), following the manufacturer's instructions. The digested products were analyzed on 1.7% agarose gel by electrophoresis stained with REALSAFE Nucleic Acid Staining®.

In a normal *β*-globin gene, generate four fragments with similar size (56 bp and 54 bp), seen as one band in the gel. In a heterozygote, sickle cell is generated in 3 fragments (110 bp, 56 bp, and 54 bp), seen as only two bands in the gel. In a homozygote, sickle cell, the enzyme, does not make any cut, and the DNA fragment remain the same.

#### 2.4.3. Haplotype Determination

Polymerase Chain Reaction-Restriction Fragment Length Polymorphism (PCR-RFLP).

Four restriction sites at the *β*-globin gene cluster were analyzed to determine haplotypes related to the HbS mutation through four restriction enzymes: *Xmn*I (5′ G*γ*), *Hind*III (G*γ*), *Hind*III (A*γ*), and *Hinf*I (5′ *β*). Each restriction fragment was amplified employing the previously described corresponding primer pair [31].

The reactions were carried out in a final volume of 25 *µ*l containing 2 *µ*l of DNA, 0.4 *µ*M of each primer, 250 *µ*M of each dNTP, and 0.625 U of Taq DNA polymerase (VWR). The amplifications were performed according to a program of 30 cycles with the following hybridization temperatures: 53°C for the fragment related to *Xmn*I, 60°C to both *Hind*III, and 55°C to *Hinf*I (Table 1).

Before digestion, the amplification products were verified by electrophoresis in a 1.5% agarose gel, stained with REALSAFE Nucleic Acid Staining Solution (Real Laboratory), and visualized by UV transillumination.

Digestions of the amplified products related to *Xmn*I and *Hinf*I were carried out in a final volume of 15 *µ*l, containing 4 *µ*l of PCR product and 0.75 *µ*l of the corresponding enzyme (Fast Digest, Thermo Fisher Scientific). Digestions of the amplification products related to *Hind*III were performed in a final volume of 20 *µ*l, including 5 *µ*l of PCR product and 0.6 *µ*l of enzyme (Promega). All digestions were carried out at 37°C and subsequently inactivated at 65°C (Table 1).

### 2.5. Statistical Analysis

Statistical analyses were conducted using *R Program for Statistical Computing, version 4.1.0* (2021). Descriptive analysis included absolute and relative frequencies for qualitative variables and median with interquartile range (IQR) for quantitative variables. In addition, regression models were employed to investigate the potential influence of HbF levels on both clinical and laboratory outcomes.

For the analysis of binary outcomes, simple and multiple logistic regression models were employed, while for continuous outcomes, simple and multiple linear regression models were used. Residual analysis was conducted to assess the adequacy of the models and to check for any violations of the regression assumptions. In addition, multicollinearity was evaluated using the variance inflation factor (VIF) to ensure that the independent variables were not highly correlated (Supplementary Materials).

### 2.6. Ethical Considerations

This prospective study was approved by the National Health Research Ethics Committee. Participation in the study was voluntary, and individuals provided informed consent by signing the informed consent form (ICF). For minors, the ICF was signed by parents or guardians.

## 3. Results

**Figure 1:**
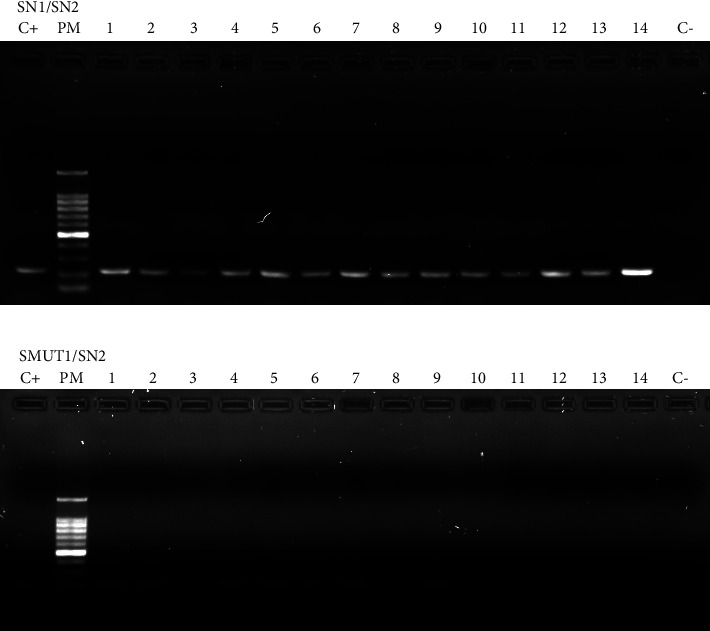
(a) Result in 1% agarose gel of ARM-PCR with SN1/SN2 primer, seen under UV light. Lane 1: C+ positive control. Lane 2 PM: 100 bp DNA ladder. Lanes 3–16: all samples. Lane 11: negative control. All samples from number 1 to 14 (lanes 2–16) show PCR amplification with SN1/SN2 beta globin primers. (b) Result in 1% agarose gel of ARM-PCR with SMUT1/SN2 beta globin primer, seen under UV light. Lane 1: C+ positive control. Lane 2 PM: 100 bp DNA ladder. Lanes 3–13 (samples 1–11) show amplification with SMUT1/SN2, meaning that the infants are carriers of SCA. Lanes 14–16 (samples 12–14) show no amplification, meaning that they are negative for SCA. Lane 17: C- negative control.

Using the HPLC technique, the presence of HbS was detected in 21 newborns (6%). Through ARMS-PCR, positive and negative samples for HbS by HPLC exhibited amplification with the normal primer for *ß*-globin, SN1 (Figure 1(a)). Conversely, only positive samples for HbS demonstrated amplification with the mutant primer for *ß*-globin, SMUT1 (Figure 1(b)). This indicates that all these infants are heterozygous for SCA.

**Figure 2:**
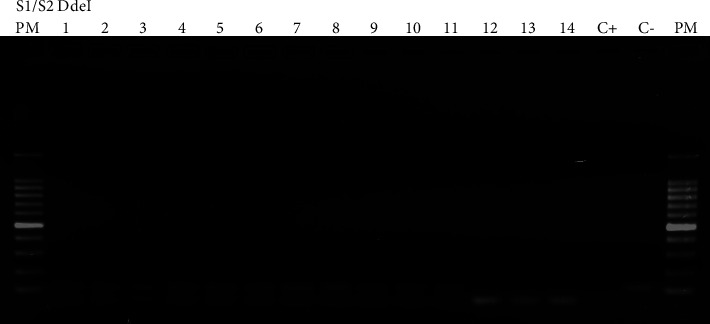
Results of gel electrophoresis of PCR-RFLP products amplified with beta-globin primers and digested with DdeI enzyme, visualized under UV light. Lane 1: 100 bp DNA ladder. Lanes 2–15: samples amplified with S1/S2 beta-globin primers and digested with DdeI enzyme. Lane 16: positive control. Lane 17: negative control. Lane 18: 100 bp DNA ladder. Interpretation: samples 1–11 exhibit amplification with beta-globin primers and partial digestion with DdeI enzyme. Samples 12, 13, and 14 show amplification with beta-globin primers and complete digestion with DdeI enzyme, indicating that the infants are negative for SCA.

All positive samples for HbS detected by HPLC revealed two bands on the gel, measuring 110 bp and 54–56 bp, upon digestion by the DdeI enzyme (Figure 2), indicating that they are heterozygotes. Infants with a normal hemoglobin profile (HbAA) exhibited a single band on the electrophoresis gel, measuring 54–56 bp.

Out of 21 newborns with sickle cell trait, it was possible to determine the haplotypes in only 15 individuals. Among these newborns, four haplotypes were distinguished: Senegal (66.6%, 10), Arabi-Indian (20%, 3), Bantu (6.7%, 1), and Benin (6.7%, 1).

Among 33 SCA patients, it was possible to determine the haplotypes in 31 individuals. In this group, most haplotypes occurred in compound heterozygosis. The Senegal/Benin haplotype was the most prevalent at 43.4% (14). Homozygous haplotypes were found for Senegal at 30.1% (10) and Benin at 6% (2). Neither Cameroon nor Arabi-Indian haplotypes were found in this group.

The median or mean of age, sex, hematological values, and selected clinical information for different haplotypes among SCA patients are presented in Table 2.

Although no statistically significant differences were found among the different haplotype groups for the analyzed parameter, we could highlight that all the cases studied present HbF values higher than 4% (Table 2).

Through multiple and simple linear regression analyses between HbF and hematological values, a significant statistical association was observed with Hb, mean concentration of hemoglobin (MCH), red cell distribution width (RDW), and white blood cells (WBCs) (Table 3). Higher levels of HbF were associated with increased levels of Hb in multiple linear regression analyses adjusted for sex and age. MCH exhibited a positive association with HbF levels in both, multiple and simple linear regression analyses. Conversely, RDW and WBC showed a negative association with HbF in both, multiple and simple linear regression analyses (Table 3 and Supplementary Tables 2, 4, 5, and 7). In other words, higher levels of HbF are associated with significant reduction of RDW and WBC. Logistic regression analysis revealed no significant relation between HbF and leg ulcers (Table 4 and Supplementary Table 10).

## 4. Discussion

High-income countries that implemented newborn screening (NBS) programs for SCA showed an effective way to reduce hospitalizations and complications in children with SCA [32]. However, the NBS program for SCA is still a challenging step to achieve in many developing countries. In this study, the diagnosis age of SCA of patients was elevated and the mean was around 10 years old. This result may reflect the lack of an NBS program for SCA in Cape Verde, and a similar result was verified in another study in Brazil [33].

In this newborn screening, from the 346 samples analyzed, 6% had sickle cell trait. This result is similar to another previous study carried out in the Santiago Island [17]. Worldwide, the prevalence of SCA differs between regions. A study made by Peil et al. (2013) predicted allele frequency higher than 15% in regions of Angola, Nigeria, and Gabon. Other countries such as Democratic Republic of Congo, Senegal, India, Saudi Arabia, and Madagascar presented allele frequency between 7.5% and 12.5% [34]. The result found in Cape Verde is similar with some regions in Brazil. This similarity may be the outcome of the settlement process, marked by slavery trade in Africa by European people, which resulted in highly miscegenated people in both countries [10, 11, 35]. The identification of sickle cell trait has important clinical significance. Although they are generally asymptomatic people when exposed to certain conditions, such as extreme temperatures, dehydration, and intense exercise, individuals with sickle cell trait can develop complications [36]. In genetic counseling, premarital diagnosis plays an important role in avoiding at-risk couples [37].

Beta S-globin haplotype determination is useful for prognostic purposes, as it helps predict the HbF level and study the genetic origin of the HbS gene in the population [19–21]. In this study, haplotype determination included both sickle cell trait and SCA patients. Among newborns, only sickle cell trait was found, with four determined haplotypes: Senegal (66.6%), Arabi-Indian (20%), Benin (6.7%), and Bantu (6.7%). In SCA patients, three haplotypes were found, occurring in homozygosis: Senegal/Senegal (30.1%) and Benin/Benin (6%), and in heterozygosis: Senegal/Benin (43.4%) and Senegal/Bantu (15.5%).

This diversity and frequency of haplotypes in the population may be a reflection of the settlement process in Cape Verde [10, 11]. The colonization process of Cape Verde began on the island of Santiago, with Africans and Europeans. The settlers were mainly black Africans, captured as slaves on the coast of Guinea, a region that included, in addition to Cape Verde, Gambia, Senegal, Guinea-Bissau, Guinea, Sierra Leone, and Liberia [38–40]. This fact is supported by the study carried out by Beleza et al. (2012), where they found that more than 57% of Santiago's ancestry came from people from West Africa [11]. This evidence may explain the predominance of Senegal haplotypes, followed by the Benin haplotype, found in this study [41].

Among the Europeans, the inhabitants were mainly Portuguese, as were the colonizers, Spanish and Italian. The Sephardic Jewish people of North Africa were another group of people who migrated to Cape Verde, especially for economic reasons [11, 38, 42]. The presence of Jews in Cape Verde may explain the frequency of the Arabi-Indian haplotypes found in this study. The Bantu haplotype showed less representation, which may reflect less influence from people from other African regions during settlement in Cape Verde [11, 41].

Regarding the hematological values found in the patients in this study, the results are in line with those described in the literature for patients with SCA. Low erythrocyte and Hb values result from the hemolytic nature of SCA, which is not accompanied by the same replacement rate [43, 44]. The median leukocyte count was high, reflecting the inflammatory status of SCA. Regarding HbF levels, elevated values were found in most patients, with a median around 11.1%, which is a common characteristic of SCA patients.

Although different classes of haplotypes are associated with clinical phenotypes of SCA patients, no statistically significant association with hematological values or HbF level was verified in this study. Regarding hematological values, similar results were found in previous studies conducted in India and Brazil [45]. These results may be due to the limited number of samples in this study, which may reduce the statistical power of the data [46]. Regrettably, for the present study, the sample size could not be increased further due to resource constraints.

However, regarding the relationship between HbF levels and hematological values, a significant association was verified with Hb, RDW, HCM, and WBC. Patients with high levels of HbF had higher levels of Hb and HCM and reduced levels of RDW, reflecting the reduction in the degree of hemolysis due to the antipolymerization effect of HbF in SCA [47]. The reduction of the RDW value may reflect the decrease in reticulocytes in the bloodstream as a result of reduced hemolysis, which underlies the pathophysiology of this disease [47]. The significant association between higher HbF and decreased leukocyte counts indicates an improvement in the inflammatory state of SCA, which plays an important role in the clinical complications of this disease [48]. Finally, although a significant association between HbF and leg ulcer was not verified, the positive impact on hematological values supports the fact that HbF is the main modulator of SCA, reducing the severity of the disease [49, 50].

## 5. Conclusions

Our study demonstrates the feasibility of implementing a neonatal screening program for early diagnosis of SCA in Cape Verde. The 6% prevalence of sickle cell trait and the late age of diagnosis of SCA patients found in this study indicate that NBS should be a priority in Cape Verde. The haplotype diversity and predominance of haplotypes from Senegal followed by Benin may reflect the settlement process in Cape Verde, with people brought as slaves from the coastal region of West Africa. Hematological values and HbF did not differ between haplotypes nor did the HbF level. However, HbF contributes to alleviate the severity of SCA, by reducing the degree of hemolysis and inflammation. Together, these parameters can be considered useful information to promote personalized medicine for patients with SCA.

## Figures and Tables

**Table 1:** Time and temperature conditions for PCR and digestion of the fragments with each restriction enzyme.

		*Xmn*I (5′ G*γ*)	*Hind*III (G*γ*)	*Hind*III (A*γ*)	*Hinf*I (5′ *β*)
PCR	Initial denaturalization	5 min at 95°C	5 min at 95°C	5 min at 95°C	5 min at 95°C
	30 cycles	45 sec at 94°C 30 sec at 53°C 45 sec at 72°C	45 sec at 94°C 30 sec at 60°C 45 sec at 72°C	45 sec at 94°C 30 sec at 60°C 45 sec at 72°C	45 sec at 94°C 30 sec at 55°C 45 sec at 72°C
	Final elongation	7 min at 72°C	7 min at 72°C	7 min at 72°C	7 min at 72°C

Digestion	Incubation	35 min at 37°C	2.5 hours at 37°C	2.5 hours at 37°C	30 min at 37°C
	Inactivation	5 min at 65°C	15 min at 65°C	15 min at 65°C	20 min at 65°C

**Table 2:** Hematological values and selected clinical complication variables in relation to SCA haplotypes.

Variable	SEN/SEN (*n* = 10)	SEN/BEN (*n* = 14)	SEN/BAT (*n* = 5)	BEN/BEN (*n* = 2)	*p* value^1^
Age^†^:	24 (13, 32)	33 (16, 37)	12 (10, 29)	18 (18, 19)	0.6
Sex^‡^:					0.8
Female	5 (50%)	6 (43%)	2 (40%)	0	
Male	5 (50%)	8 (57%)	3 (60%)	2 (100%)	
Hb (g/dL)^†^	8.4 (7.25, 9.8)	7.5 (6.67, 9.1)	8 (7.6, 9.2)	6.95 (6.72, 7.18)	0.2
RBC (^*∗*^10^9^/L)^†^	2.97 (2.65, 3.36)	2.42 (2.13, 2.72)	2.87 (2.55, 2.94)	2.48 (2.37, 2.59)	0.2
HCT (%)^†^	25.7 (21.4, 29.2)	21.6 (19.3, 27)	23 (22.7, 27)	20.7 (19.9, 21.5)	0.2
MCV (fL)^†^	85 (79, 90)	92 (88, 93)	90 (79, 92)	83 (83, 84)	0.2
MCH (g/dL)^†^	28.2 (25.8, 31.5)	31.5 (30.5, 32.6)	31.3 (25.6, 31.9)	28.1 (27.7, 28.5)	0.3
MCHC (g/dL)^†^	33.6 (32.6, 34.67)	34.4 (33.68, 34.68)	33.6 (33.5, 34.1)	33.7 (33.45, 33.95)	0.4
RDW (%)^†^	23.5 (20.5, 24.6)	23.8 (21.5, 25.3)	20 (19, 21.9)	26.4 (25.2, 27.7)	0.12
WBC (^*∗*^10^9^/L)^†^	11.5 (9.83, 12.9)	10.4 (8.85, 11.13)	11 (9.4, 11.1)	13.7 (12.55, 14.85)	0.3
Lymphocytes (^*∗*^10^9^/L)^†^	33 (30, 36)	39 (35, 44)	39 (30, 45)	36 (33, 40)	0.3
Platelets (^*∗*^10^9^/L)^†^	350 (265, 466)	392 (252, 444)	458 (451, 514)	750 (659, 842)	0.07
HbF (%)^†^	11.1 (7.3, 15)	9.7 (7.4, 12.1)	15.1 (12.4, 18.1)	4.2 (2.7, 5.7)	0.2
Stroke^‡^	0	2 (14%)	0	0	0.7
Priapism^‡^	1 (10%)	3 (21%)	0	1 (50%)	0.3
Leg ulcer^‡^	6 (60%)	6 (43%)	1 (20%)	1 (50%)	0.6

Hb: hemoglobin; RBC: red blood cell; Hct: hematocrit; MCV: mean corpuscular volume; MCH: mean concentration hemoglobin; MCHC: mean concentration hemoglobin corpuscular; RDW: red cell distribution width; WBC: white blood cell; HbF: fetal hemoglobin; HbS: hemoglobin S; SEN: Senegal; BEN: Benin; BAT: Bantu. ^1^Fisher's exact test for qualitative variables; Kruskal–Wallis rank sum test for quantitative variables. ^†^Values are expressed as median and interquartile range (IQR). ^‡^Values are expressed in *n* (%).

**Table 3:** Simple and multiple linear regression of HbF with hematological variables of patients.

Description	*N* = 33	Simple			Multiple^1^		
		Beta	95% CI	*p* value	Beta	95% CI	*p* value
RBC (10^6^/mL)^†^	2.58 (2.38, 3.00)	0.01	−0.04, 0.05	0.798	0.01	−0.03, 0.05	0.656
Hb (g/dL)^†^	7.90 (7.00, 9.20)	0.08	−0.01, 0.17	0.065	0.09	0.01, 0.17	0.022
Hct (%)^†^	22.7 (20.6, 27.4)	0.31	0.22, 1.40	0.171	0.27	0.126, 1.8	0.081
MHC (pg)^†^	30.9 (27.4, 31.9)	0.24	0.02, 0.46	0.031	0.25	0.02, 0.47	0.031
MCHC (g/dL)^†^	33.9 (33.2, 34.6)	0.06	−0.01, 0.12	0.075	0.06	−0.01, 0.12	0.088
RDW (%)^†^	23.0 (20.1, 24.8)	−0.42	−0.58, −0.26	<0.001	−0.43	−0.59, −0.27	<0.001
WBC (10^3^/mL)^†^	11.1 (9.4, 12.9)	−0.16	−0.30, −0.01	0.033	−0.17	−0.31, −0.02	0.024
Lymphocytes (%)^†^	36 (31, 43)	0.41	−0.13, 0.95	0.135	0.37	−0.17, 0.90	0.128
Platelets (10^3^/mL)^†^	437 (257, 494)	−7.8	−18, 2.8	0.146	−9.2	−19, 0.62	0.065

CI: confidence interval; RBC: red blood cell; Hb: hemoglobin; Hct: hematocrit; MCH: mean concentration hemoglobin; MCHC: mean concentration hemoglobin corpuscular; RDW: red cell distribution width; WBC: white blood cell. ^1^Adjusted variables: sex and age. ^†^Values are expressed as median and interquartile range (IQR).

**Table 4:** Simple and multiple logistic regression of HbF with leg ulcer of patients with SCA.

Description	*N* = 33	Simple			Multiple^1^		
		OR	95% CI	*p* value	OR	95% CI	*p* value
Leg ulcer^†^	15 (45%)	−0.7	−0.21, 0.06	0.263	−0.07	−0.21, 0.06	0.307

OR: odds ratio; CI: confidence interval. ^1^Adjusted variables: age and sex. ^†^Values are expressed in *n* (%).

## Data Availability

The data that support the findings of this study are available from the corresponding author on request. The data are not publicly available due to privacy or ethical restrictions.

## Supplementary Materials

Supplementary Materials Supplementary materials provide additional information on the regression analysis of fetal hemoglobin (HbF) with hematological variables and clinical characteristic. It is organized in sections numerated from 1 to 10, and each section is titled accordingly with corresponding dependent variables. The sections contain tables (Supplementary Tables 1–10) that present results of the simple and multiple regression analysis, as well as the verification of assumptions by means of tests and graphs (Supplementary Figures 1–9).
