# Supplementary material for: Sickle Cell Anemia Screening in Newborns and Analysis of Haplotypes in Patients from Santiago Island, Cape Verde
Source: Anemia. 2024 Aug 27;2024:1687917. doi: 10.1155/2024/1687917 (PMC11371444; doi:10.1155/2024/1687917)
Supplement: Supplementary Materials — provide additional information on the regression analysis of fetal hemoglobin (HbF) with hematological variables and clinical characteristic. It is organized in sections numerated from 1 to 10, and each section is titled accordingly with corresponding dependent variables. The sections contain tables (Supplementary Tables 1–10) that present results of the simple and multiple regression analysis, as well as the verification of assumptions by means of tests and graphs (Supplementary Figures 1–9). [file 1687917.f1.docx]

S**imple and multiple regression analysis of Fetal Hemoglobin (HbF) with hematological variables and clinical characteristic:**

***1- Red Blood Cells (RBC)***

**Table 1. Simple and multiple linear regression of Fetal Hemoglobin (HbF) with Red Blood Cells (RBC)**

|  | *Multiple* | | | *Simple* | | |
| --- | --- | --- | --- | --- | --- | --- |
| Characteristic | Beta | 95% CI^1^ | p-value | Beta | 95% CI^1^ | p-value |
| HbF | 0.01 | -0.03, 0.05 | 0.656 | 0.01 | -0.04, 0.05 | 0.798 |
| Age | 0.01 | -0.01, 0.03 | 0.166 |  |  |  |
| Sex |  |  |  |  |  |  |
| Female | — | — |  |  |  |  |
| Male | 0.30 | -0.17, 0.77 | 0.200 |  |  |  |

^1^CI = Confidence Interval

Variance Inflation Factor (VIF) for all independent variables is below 2, suggesting no multicollinearity problems. Residuals analysis shows no problems (Shapiro-Wilk teste: $p=0.1091$; Durbin-Watson test: $p=0.9936$; Breusch-Pagan test: $p=0.2955$).


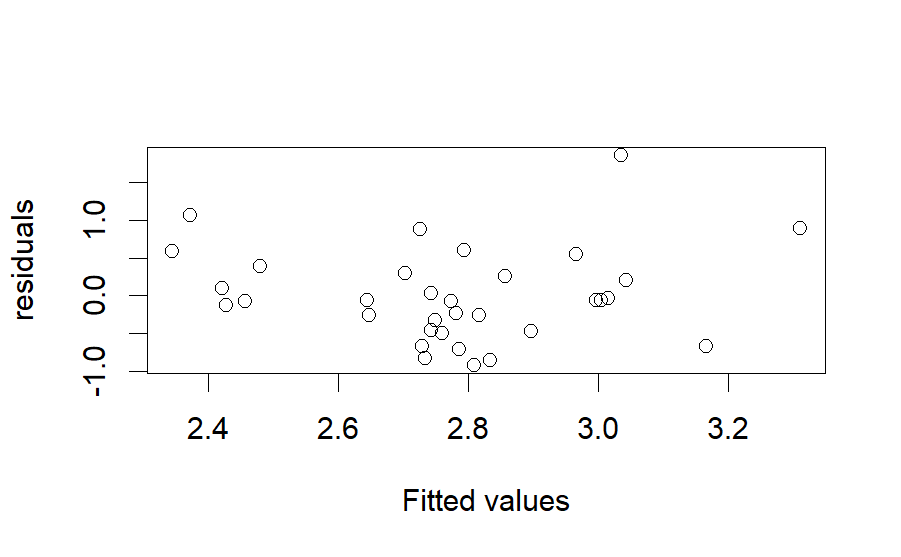


**Figure 1- Residuals vs Fitted plot**

***2- Hemoglobin (Hb)***

**Table 2. Simple and multiple linear regression of Fetal Hemoglobin (HbF) with Hemoglobin (Hb)**

|  | *Multiple* | | | *Simple* | | |
| --- | --- | --- | --- | --- | --- | --- |
| Characteristic | Beta | 95% CI^1^ | p-value | Beta | 95% CI^1^ | p-value |
| HbF | 0.09 | 0.01, 0.17 | **0.022** | 0.08 | -0.01, 0.17 | 0.065 |
| Age | 0.04 | 0.00, 0.07 | **0.039** |  |  |  |
| Sex |  |  |  |  |  |  |
| Female | — | — |  |  |  |  |
| Male | 1.0 | 0.07, 1.9 | **0.035** |  |  |  |

^1^CI = Confidence Interval

Variance Inflation Factor (VIF) for all independent variables is below 2, suggesting no multicollinearity problems. Residuals analysis shows no problems (Shapiro-Wilk teste: $p=0.6182$; Durbin-Watson test: $p=0.9663$; Breusch-Pagan test: $p=0.5556$).


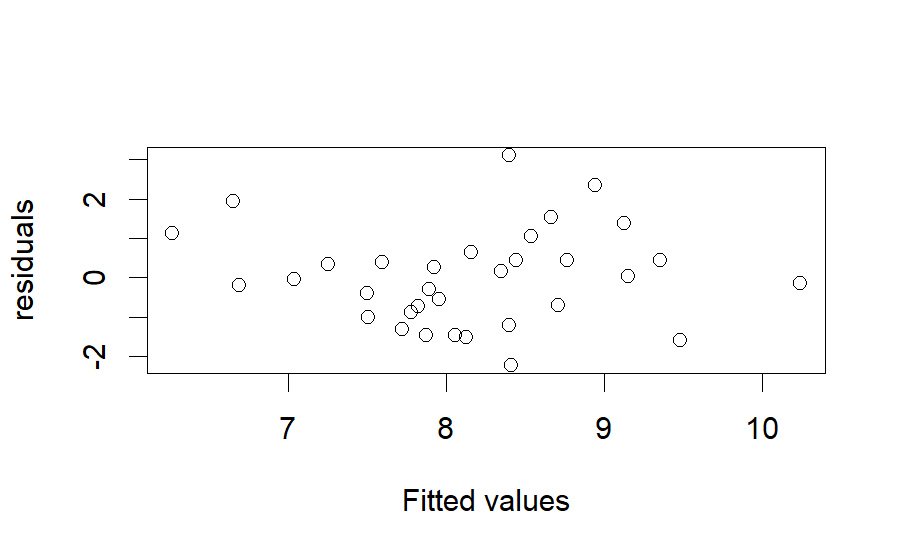


**Figure 2- Residuals vs Fitted**

***3- Hematocrit (Hct)***

**Table 3. Simple and multiple linear regression of Fetal Hemoglobin (HbF) with Hematocrit (Hct)**

|  | *Multiple* | | | | *Simple* | | |
| --- | --- | --- | --- | --- | --- | --- | --- |
| Characteristic | Beta | 95% CI^1^ | p-value | | Beta | 95% CI^1^ | p-value |
| HbF | 0.23 | -0.03, 0.48 | | 0.081 | 0.19 | -0.09, 0.47 | 0.171 |
| Age | 0.12 | 0.00, 0.23 | **0.047** | |  |  |  |
| Sex |  |  |  | |  |  |  |
| Female | — | — |  | |  |  |  |
| Male | 2.9 | -0.05, 5.9 | 0.053 | |  |  |  |

^1^CI = Confidence Interval

Variance Inflation Factor (VIF) for all independent variables is below 2, suggesting no multicollinearity problems. Residuals analysis shows no problems (Shapiro-Wilk teste: $p=0.4118$; Durbin-Watson test: $p=0.9938$; Breusch-Pagan test: $p=0.4744$).


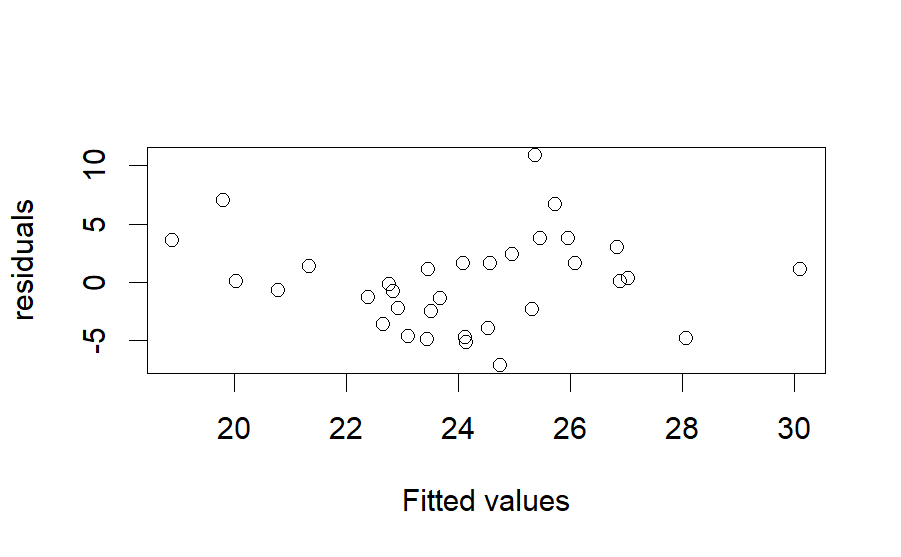


**Figure 3- Residuals vs Fitted**

***4-*** ***Red Blood Cells distribution width (RDW)***

**Table 4. Simple and multiple linear regression of Fetal Hemoglobin (HbF) with Red Blood Cells distribution width (RDW).**

|  | *Multiple* | | | | *Simple* | | |
| --- | --- | --- | --- | --- | --- | --- | --- |
| Characteristic | Beta | 95% CI^1^ | p-value | | Beta | 95% CI^1^ | p-value |
| HbF | -0.43 | -0.59, -0.27 | | **<0.001** | -0.42 | -0.58, -0.26 | **<0.001** |
| Age | -0.05 | -0.12, 0.03 | 0.196 | |  |  |  |
| Sex |  |  |  | |  |  |  |
| Female | — | — |  | |  |  |  |
| Male | 0.31 | -1.5, 2.2 | 0.736 | |  |  |  |

^1^CI = Confidence Interval

Variance Inflation Factor (VIF) for all independent variables is below 2, suggesting no multicollinearity problems. Residuals analysis shows no problems (Shapiro-Wilk teste: $p=0.2613$; Durbin-Watson test: $p=0.7353$; Breusch-Pagan test: $p=0.08642$).


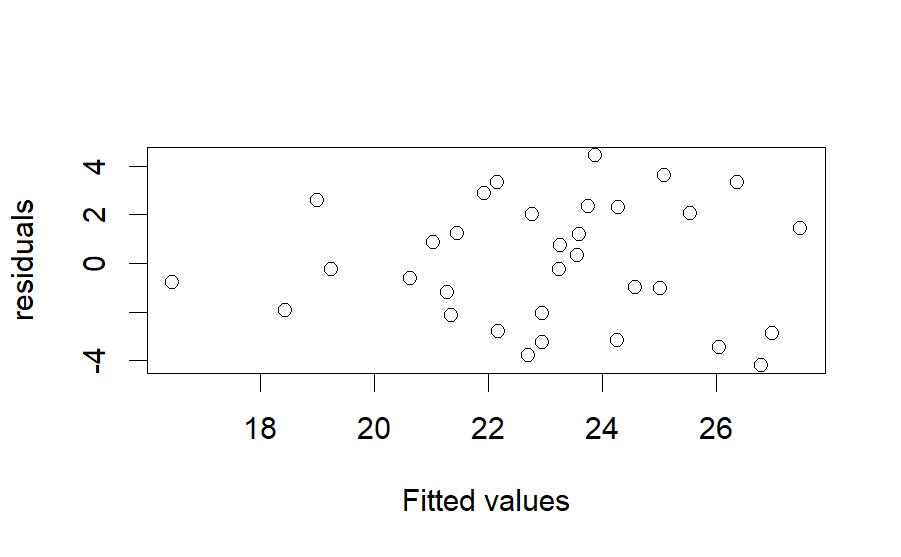


**Figure 4- Residuals vs Fitted**

***5- Mean Corpuscular Hemoglobin (MCH)***

**Table 5. Simple and multiple linear regression of Fetal Hemoglobin (HbF) with Mean Corpuscular Hemoglobin (MCH).**

|  | *Multiple* | | | | *Simple* | | |
| --- | --- | --- | --- | --- | --- | --- | --- |
| Characteristic | Beta | 95% CI^1^ | p-value | | Beta | 95% CI^1^ | p-value |
| HbF | 0.25 | 0.02, 0.47 | | **0.031** | 0.24 | 0.02, 0.46 | **0.031** |
| Age | 0.02 | -0.08, 0.12 | 0.674 | |  |  |  |
| Sex |  |  |  | |  |  |  |
| Female | — | — |  | |  |  |  |
| Male | 0.91 | -1.7, 3.5 | 0.479 | |  |  |  |

^1^CI = Confidence Interval

Variance Inflation Factor (VIF) for all independent variables is below 2, suggesting no multicollinearity problems. Residuals analysis shows no problems (Shapiro-Wilk teste: $p=0.8755$; Durbin-Watson test: $p=0.6017$; Breusch-Pagan test: $p=0.07953$).


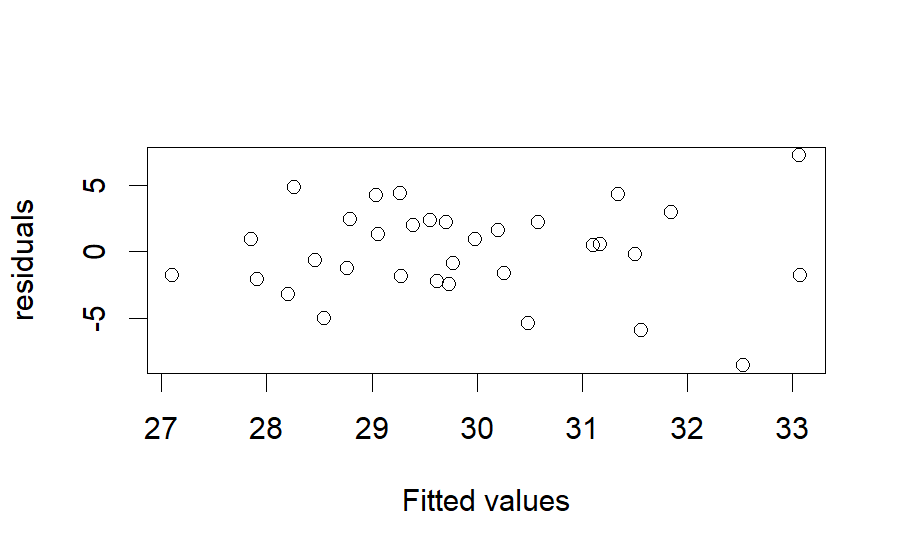


**Figure 5- Residuals vs Fitted**

***6- Mean Corpuscular Hemoglobin Concentration (MCHC)***

**Table 6. Simple and multiple linear regression of Fetal Hemoglobin (HbF) with Mean Corpuscular Hemoglobin Concentration (MCHC)**

|  | *Multiple* | | | | | *Simple* | | |
| --- | --- | --- | --- | --- | --- | --- | --- | --- |
| Characteristic | Beta | 95% CI^1^ | p-value | | | Beta | 95% CI^1^ | p-value |
| HbF | 0.06 | -0.01, 0.12 | | 0.088 | | 0.06 | -0.01, 0.12 | 0.075 |
| Age | 0.00 | -0.03, 0.03 | | 0.867 | |  |  |  |
| Sex |  |  | | |  |  |  |  |
| Female | — | — |  | | |  |  |  |
| Male | 0.12 | -0.66, 0.89 | 0.763 | | |  |  |  |

^1^CI = Confidence Interval

Variance Inflation Factor (VIF) for all independent variables is below 2, suggesting no multicollinearity problems. Residuals analysis shows no problems (Shapiro-Wilk teste: $p=0.2793$; Durbin-Watson test: $p=0.9415$; Breusch-Pagan test: $p=0.4052$).


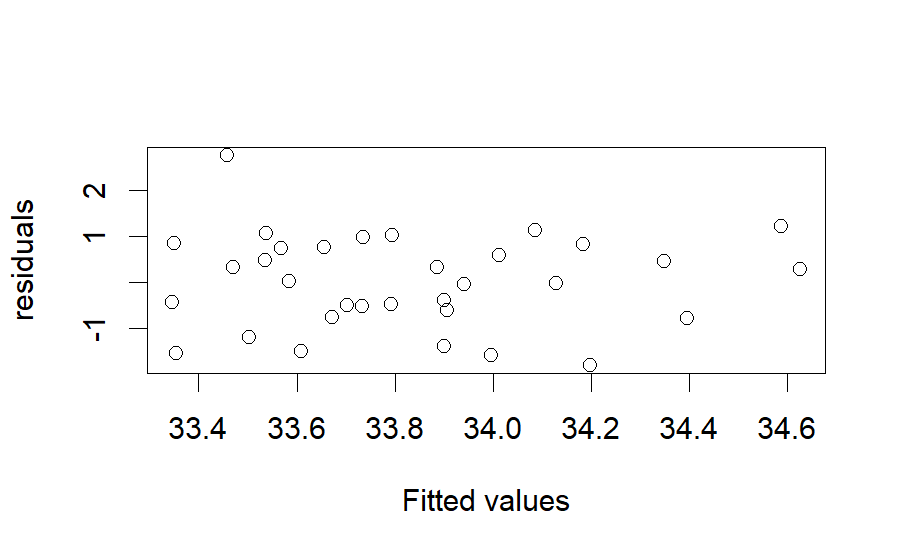


**Figure 6- Residuals vs Fitted**

***7- White Blood Cells (WBC)***

**Table 7. Simple and multiple linear regression of Fetal Hemoglobin (HbF) with White Blood Cells (WBC)**

|  | *Multiple* | | | | | *Simple* | | |
| --- | --- | --- | --- | --- | --- | --- | --- | --- |
| Characteristic | Beta | 95% CI^1^ | p-value | | | Beta | 95% CI^1^ | p-value |
| HbF | -0.17 | -0.31, -0.02 | | **0.024** | | -0.16 | -0.30, -0.01 | **0.033** |
| Age | -0.05 | -0.11, 0.02 | | 0.155 | |  |  |  |
| Sex |  |  | | |  |  |  |  |
| Female | — | — |  | | |  |  |  |
| Male | 0.92 | -0.72, 2.6 | 0.261 | | |  |  |  |

^1^CI = Confidence Interval

Variance Inflation Factor (VIF) for all independent variables is below 2, suggesting no multicollinearity problems. Residuals analysis shows no problems (Shapiro-Wilk teste: $p=0.27$; Durbin-Watson test: $p=0.5989$; Breusch-Pagan test: $p=0.2693$).


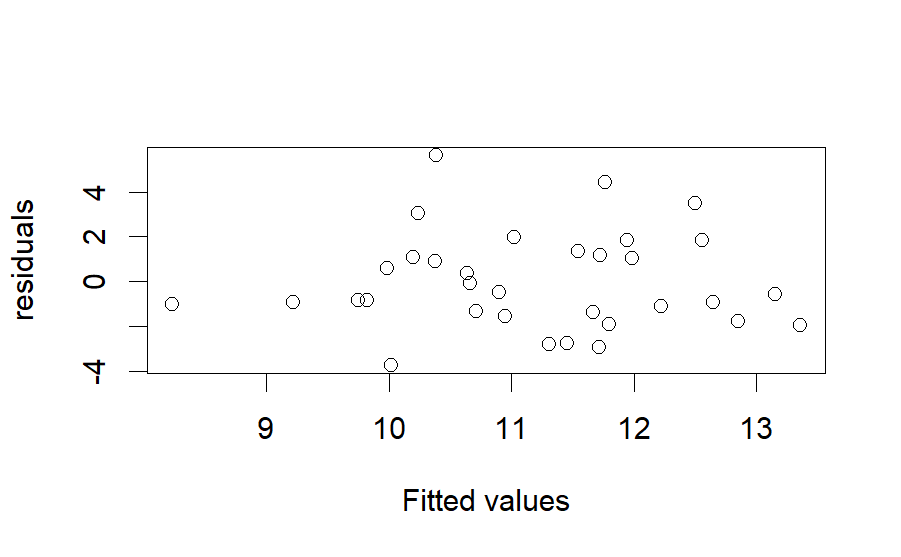


**Figure 7- Residuals vs Fitted**

***8-*** ***Lymphocytes***

**Table 8. Simple and multiple linear regression of Fetal Hemoglobin (HbF) with Lymphocytes.**

|  | *Multiple* | | | | | *Simple* | | |
| --- | --- | --- | --- | --- | --- | --- | --- | --- |
| Characteristic | Beta | 95% CI^1^ | p-value | | | Beta | 95% CI^1^ | p-value |
| HbF | 0.37 | -0.17, 0.90 | | 0.174 | | 0.41 | -0.13, 0.95 | 0.128 |
| Age | -0.18 | -0.43, 0.06 | | 0.135 | |  |  |  |
| Sex |  |  | | |  |  |  |  |
| Female | — | — |  | | |  |  |  |
| Male | -0.75 | -7.0, 5.5 | 0.806 | | |  |  |  |

^1^CI = Confidence Interval

Variance Inflation Factor (VIF) for all independent variables is below 2, suggesting no multicollinearity problems. Residuals analysis shows no problems (Shapiro-Wilk teste: $p=0.4088$; Durbin-Watson test: $p=0.2293$; Breusch-Pagan test: $p=0.2451$).


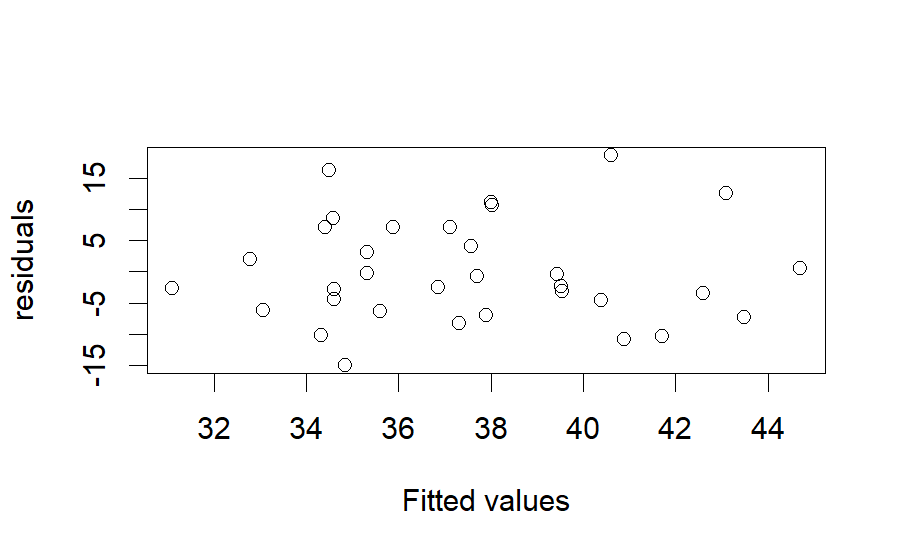


**Figure 8- Residuals vs Fitted**

***9- Platelets***

**Table 9. Simple and multiple linear regression of Fetal Hemoglobin (HbF) with Platelets**

|  | *Multiple* | | | | | *Simple* | | |
| --- | --- | --- | --- | --- | --- | --- | --- | --- |
| Characteristic | Beta | 95% CI^1^ | p-value | | | Beta | 95% CI^1^ | p-value |
| HbF | -9.2 | -19, 0.62 | | 0.065 | | -7.8 | -18, 2.8 | 0.146 |
| Age | -6.2 | -11, -1.7 | | **0.008** | |  |  |  |
| Sex |  |  | | |  |  |  |  |
| Female | — | — |  | | |  |  |  |
| Male | 18 | -95, 131 | 0.750 | | |  |  |  |

^1^CI = Confidence Interval

Variance Inflation Factor (VIF) for all independent variables is below 2, suggesting no multicollinearity problems. Residuals analysis shows no problems (Shapiro-Wilk teste: $p=0.0825$; Durbin-Watson test: $p=0.9893$; Breusch-Pagan test: $p=0.6101$).


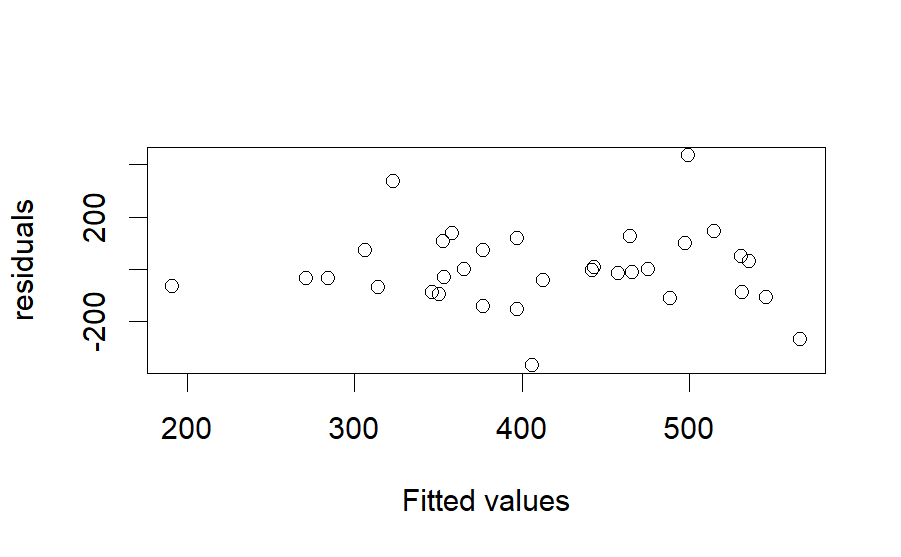


**Figure 9- Residuals vs Fitted**

***10- Leg Ulcer***

**Table 10. Simple and multiple logistic regression of Fetal Hemoglobin (HbF) with Leg Ulcer**

|  | *Multiple* | | | | | *Simple* | | |
| --- | --- | --- | --- | --- | --- | --- | --- | --- |
| Characteristic | OR^1^ | 95% CI^1^ | p-value | | | OR^1^ | 95% CI^1^ | p-value |
| HbF | -0.07 | -0.21, 0.06 | | 0.307 | | -0.07 | -0.21, 0.05 | 0.263 |
| Age | 0.03 | -0.02, 0.09 | | 0.287 | |  |  |  |
| Sex |  |  | | |  |  |  |  |
| Female | — | — |  | | |  |  |  |
| Male | 0.16 | -1.3, 1.6 | 0.824 | | |  |  |  |

^1^OR = Odds Ratio, CI = Confidence Interval

Variance Inflation Factor (VIF) for all independent variables is below 2, suggesting no multicollinearity problems.

The Wilcoxon Mann-Whitney test was also used to test the association between HbF and the occurrence of Priapism. No significant association was found ($p=0.2121$), converging on the same result found in logistic regression.
